# Circulating miR-483-3p and miR-21 is highly expressed in plasma of pancreatic cancer

**DOI:** 10.3892/ijo.2014.2743

**Published:** 2014-11-10

**Authors:** MAKOTO ABUE, MISA YOKOYAMA, RIE SHIBUYA, KEIICHI TAMAI, KAZUNORI YAMAGUCHI, IKURO SATO, NOBUYUKI TANAKA, SHIN HAMADA, TOORU SHIMOSEGAWA, KAZUO SUGAMURA, KENNICHI SATOH

**Affiliations:** 1 Division of Cancer Stem Cell, Miyagi Cancer Center Research Institute, Medeshima Siote, Natori, Miyagi 981-1293, Japan; 2 Division of Cancer Biology and Therapeutics, Miyagi Cancer Center Research Institute, Medeshima Siote, Natori, Miyagi 981-1293, Japan; 3 Division of Molecular and Cellular Oncology, Miyagi Cancer Center Research Institute, Medeshima Siote, Natori, Miyagi 981-1293, Japan; 4 Department of Pathology, Miyagi Cancer Center, Medeshima Siote, Natori, Miyagi 981-1293, Japan; 5 Division of Gastroenterology, Tohoku University Graduate School of Medicine, Aoba-ku, Sendai, Miyagi 980-8574, Japan

**Keywords:** plasma, miR-483-3p, miR-21, pancreatic cancer, biomarker

## Abstract

Several recent studies have revealed that microRNAs (miRNAs) have a role in carcinogenesis and cancer development, and that it is stably detectable in plasma/serum. The aim of this study was to test whether miR-483-3p as well as miR-21 could be plasma biomarkers for PDAC. The plasma samples were obtained from three groups including 32 pancreatic ductal adenocarcinoma (PDAC) patients, 12 patients with intraductal papillary mucinous neoplasm (IPMN) patients and 30 healthy controls (HC). We evaluated the plasma miR-483-3p and miR-21 expression level by quantitative RT-PCR. We compared the differences in the plasma level of these miRNAs among the three groups, and investigated the relevance of their plasma expression level to the clinical factors in PDAC. The expressions of miR-483-3p and miR-21 were detected in all examined plasma samples. The plasma expression levels of these miRNAs were significantly higher in PDAC compared to HC (P<0.01). The plasma miR-483-3p expression was significantly higher in PDAC patients than IPMN patients (P<0.05). The plasma miR-21 level was associated with advanced stage (P<0.05), metastasis to lymph node and liver (P<0.01), and shorter survival (P<0.01) of the PDAC patients. Together, these findings suggest that measurement of the plasma miR-483-3p level is useful for discriminating PDAC from IPMN, and that the plasma miR-21 level predicts outcome of PDAC patients.

## Introduction

Pancreatic ductal adenocarcinoma (PDAC) is the fifth leading cause of cancer death in Japan (1). This disease usually shows a poor prognosis because of the rapid progression and development of distant metastasis by the time of diagnosis (2,3). In addition, this tumor is resistant to conventional chemotherapy and radiation therapy. Effective technologies such as positron emission tomography (PET) and endoscopic ultrasound-guided fine needle aspiration-biopsy (EUS-FNAB) can improve the detection of PDAC. However, the availability of these methods is restricted due to their high costs or difficulty of technique. On the other hand, the serum levels of carcinoembryonic antigen (CEA) and carbohydrate antigen 19-9 (CA19-9) have been used as useful markers for the presence of PDAC (4). However, their sensitivity and specificity for the early detection of PDAC is not sufficient. Therefore, an easy and accurate method for the detection of PDAC is required to improve its poor prognosis.

MicroRNAs (miRNAs) are small non-coding RNAs composed of 18–25 nucleotides that regulate the translation of specific genes through binding to the 3′-untranslated regions (UTRs) of their target mRNAs. Since first discovered by Lee *et al* in 1993 (5), accumulating evidence has revealed that miRNAs play important roles in the generation or development of various cancers. In PDAC tissues, a number of studies have identified multiple aberrantly expressed miRNAs, including miR-21 (6–9), miR-155 (7–9), miR-146a (10), miR-196a (11), miR-196b (12), miR-200a/b/c (13–15), miR-221 and miR-222 (7,16). Interestingly, miRNAs are stably detectable in the plasma/serum since they are protected from RNase activity (17–20) by microvesicles like exosomes (21–23), forming protein complexes with Ago2 (24), and lipoprotein complexes (25). The stability of miRNAs in body fluids suggests that circulating miRNAs could be useful diagnostic markers in various cancers. Recently, it has been reported that increased or decreased expression of circulating miRNAs, such as miR-18a, miR-21, miR-155, miR-196a, and miR-200a/b, miR-210, miR-221, are correlated with the development of PDAC (20,26–29) (Table I). Among these miRNAs, the correlation between tumor progression and the expression of miR-21 has been well documented in PDAC patients (26,30–37).

We previously performed comprehensive analysis of miRNA expression profiles in PDAC and non-invasive intraductal papillary-mucinous neoplasm (IPMN) (GSE29542) to explore the miRNAs involved in the invasive growth of PDAC. We identified miR-126 and miR-197 as differentially expressed miRNAs in PDAC and revealed that these miRNAs contributed to the invasion of PDAC cells (38,39). We also found miR-483-3p as one of the upregulated miRNAs along with miR-21 and-197 in PDAC by re-reviewing this comprehensive analysis. miR-483-3p, which is located within intron 2 of the IGF2 locus, is overexpressed in a variety of tumors such as malignant mesothelioma, Wilms’ tumor tissues, colon, breast and liver cancers (40,41). In addition, the expression of miR-483-3p is strongly enhanced in PDAC tissues and suppresses the expression of DPC4/Smad4 (42). However, little is known about the plasma expression of miR-483-3p and its association with the clinicopathological features in PDAC patients. Therefore, we tested whether evaluation of the plasma miR-483-3p level would be useful for detecting PDAC, and investigated its possible use as a biomarker by comparing it with plasma miR-21 and conventional tumor marker (CEA and CA19-9) levels.

## Materials and methods

### Microdissection from tissue samples

Ten PDAC and 13 IPMN tissue samples were obtained from patients who underwent surgical resection in Miyagi Cancer Center Hospital from 2010 to 2012. Histologic diagnosis of each sample was performed by two pathologists who were not informed about the present study. Histologically, cancer duct cells and non-tumor cells (mixed with acinar cells, inflammatory duct cells and stromal cells) from PDAC tissues (n=10), and non-invasive IPMN cells (adenoma and carcinoma *in situ*, n=13) were microdissected and subjected to RNA extraction. These paraffin-embedded tissues were cut into 10-μm sections and ~10 sequential regions from the same paraffin block were microdissected using a Leica CIR MIC system (Leica Microsystems, Wetzkar, Germany). Total RNA including miRNA was extracted using the Recover All Total Nucleic Acid Isolation kit (Ambion, Austin, TX, USA), according to the manufacturer’s instructions.

### Patients and blood samples

Between April 2011 and March 2013, the plasma samples of 32 PDAC patients, 30 healthy controls (HC) and 12 IPMN patients were collected at Miyagi Cancer Center. Patient characteristics with respect to age, sex, and stages of disease are described in Table II. The stage of PDAC was assessed according to the Union for International Cancer Control (UICC) Classification (43). All patients were pathologically diagnosed as having PDAC using surgical (n=8) or biopsy specimens [EUS-FNA (n=20), endoscopic transpapillary biopsy (n=3) and biopsy from duodenal invasion (n=1)]. As a control, plasma samples were collected from 30 HC. HC were 22 medical staff members and 8 hospitalized patients with benign disease such as asymptomatic cholecystolithiasis or choledocholithiasis. They underwent medical examinations and did not have any pancreatic disease. All IPMN patients were the branch duct type of IPMNs (BD-IPMN). In this study, the definition of BD- IPMN was based on imaging as a grapelike multilocular cystic lesion without mural nodules communicating with the MPD by computed tomography (CT), magnetic resonance cholangiopancreatography (MRCP), endoscopic ultrasonography (EUS), and/or endoscopic retrograde cholangiopancreatography (ERCP).

### Blood plasma samples and RNA extraction

Blood samples were collected from patients and controls in sodium EDTA-Na tubes and were immediately centrifuged at 3500 rpm for 10 min at room temperature. Then the plasma supernatant was collected and stored at −80°C until further analysis. Total RNA containing small RNA was extracted from 300 μl plasma samples using a mirVana PARIS kit (Ambion), undiluted into 100 μl of preheated (95°C) elution solution according to the manufacturer’s instructions. To normalize sample-to-sample variation in the RNA isolation step, 2 μl of synthetic *C. elegans* miR-39, which lacks sequence homology to human miRNAs, was added to each denatured sample.

### The detection of miRNAs

The amount of miRNAs was quantified in duplicate via quantitative RT-PCR using the human TaqMan MicroRNA Assay kit (Applied Biosystems, Foster City, CA, USA). The reverse transcription reaction was carried out with a TaqMan MicroRNA Reverse Transcription kit (Applied Biosystems) in 15 μl containing 5 μl of RNA extract, 0.15 μl of 100 mM dNTPs, 1.5 μl of 10× reverse transcriptase buffer, 1 μl of Multiscribe reverse transcriptase (50 U/μl), 0.19 μl of RNase inhibitor (20 U/μl), 3 μl of gene-specific primer, and 4.16 μl of Nuclease-free water. For the synthesis of cDNA, reaction mixtures were incubated at 16°C for 30 min, at 42°C for 30 min, and at 85°C for 5 min, and then held at 4°C. Next, 15 μl of cDNA solution was amplified with 25 μl of TaqMan 2× Universal PCR Master Mix with no AmpErase UNG (Applied Biosystems), 2.5 μl of gene-specific primers/ probe, and 7.5 μl of nuclease-free water in a final volume of 50 μl. Quantitative RT-PCR was run on a LightCycler^®^ 480 real-time PCR system (Roche), and reaction mixtures were incubated at 95°C for 10 min, followed by 40 cycles of 95°C for 15 sec, and 60°C for 1 min. Cycle threshold (Ct) values were calculated with the LightCycler 480 for Software Version 1.5 (Roche). The relative expression of the mature miRNAs was calculated using the comparative CT (2^−2ΔΔCt^) method with miR-16 and RNU6B for plasma and tissue samples, respectively, as the endogenous control to normalize the data (44,45).

### Statistical analysis

Student’s t-test or Mann-Whitney U test was used to evaluate differences in the miRNA expression between PDAC or IPMN cases and controls. Receiver operating characteristic (ROC) curves were constructed and the area under the curve (AUC) was calculated to evaluate the sensitivity and specificity for predicting cases and controls based on the expression of each individual miRNA and their combinations. Fisher’s exact test or Pearson’s χ^2^ test was used to determine if there was a significant association between the clinicopathological factors and the relative plasma expression levels of miRNAs. Survival rate was estimated by the Kaplan-Meier method. The difference in survival rates between the groups was tested for significance using the log-rank test. The overall survival was defined as the period from the date of diagnosis for PDAC to the date of death or last follow-up. All tests of statistical significance were two-sided. A P-value of <0.05 was considered statistically significant. All statistical analyses were done using Excel 2010 software.

### Ethics

The Institutional Review Board of the Miyagi Cancer Center (MCC) approved this study protocol, and written informed consent was obtained from each patient (permit MCC-24-38).

## Results

### The expression of miR-483-3p as well as miR-21 is enhanced in PDAC cells compared to IPMN and normal pancreatic cells

**Figure 1:**
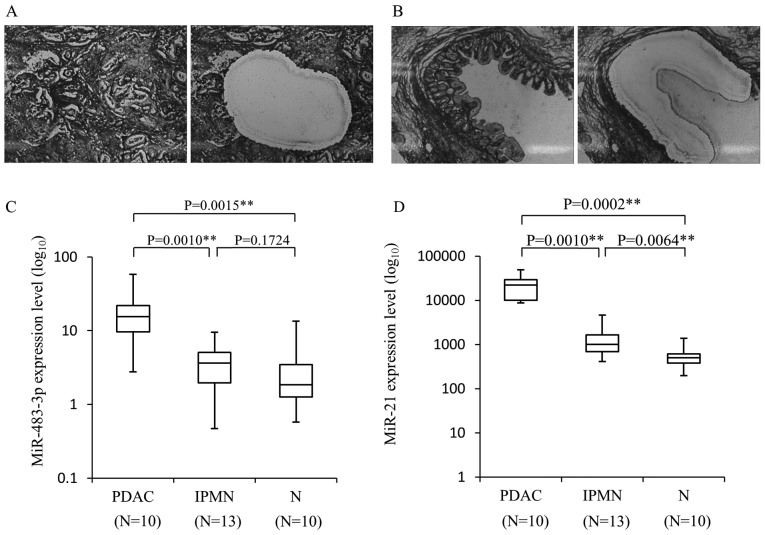
The expression of miR-483-3p and miR-21 in microdissected lesions. MicroRNA extraction was carried out from the microdissected lesions. Neoplastic lesions stained with hematoxylin [left panels in (A) and (B)] were selectively dissected [right panels in (A) and (B)] using a Leica CIR MIC system and recovered in lysis buffer. (A) PDAC tissue; (B) IPMN tissue. (C) The miRNA-483-3p and (D) miR-21 expression levels were detected by quantitative RT-PCR. Boxes represent the interquartile range and the line indicates the median value. Whiskers indicate maximum and minimum values. RNU6B was used for normalization. The miR-483-3p and miR-21 expression levels are significantly higher in PDAC tissue than in non-cancer tissue and IPMN tissue. Mann-Whitney U test was used to evaluate statistical significance. N, non-cancer tissue. ^**^P<0.01.

To validate the results of the previous comprehensive analysis, we first examined the miR-483-3p and miR-21 expression levels in a new series of PDAC and IPMN tissues. The results for these miRNAs, normalized with RNU6B are shown in Fig. 1. In microdissected PDAC cells, the mean level of miR-483-3p expression was 19.36±5.23 [miR-483-3p/RNU6B, mean ± standard error (SE)], and this expression level was significantly higher than that of non-tumor cells (3.26±1.22, P=0.0015). The miR-483-3p expression in PDAC cells was also significantly higher than in IPMN lesions (4.07±0.75, P=0.0010). Although there was no statistically significant difference between IPMN cells and non-tumor cells, the miR-483-3p level tended to be slightly higher in the IPMN samples than in the normal tissue, suggesting that the increased expression of miR-483-3p occurs in the transition from benign to malignant. Similarly, the expression of miR-21 was significantly higher in PDAC [23150.61±4493.76 (miR-21/RNU6B, mean ± SE)] than IPMN (1413.05±319.18, P=0.0001) and non-tumor cells (580.45±109.06, P=0.0002). On the other hand, miR-21 expression was significantly higher in IPMN compared to non-tumor cells, suggesting that miR-21 contributes to an early step of pancreatic tumorigenesis.

### Plasma expression level of miR-483-3p and -21 is increased in PDAC patient

**Figure 2:**
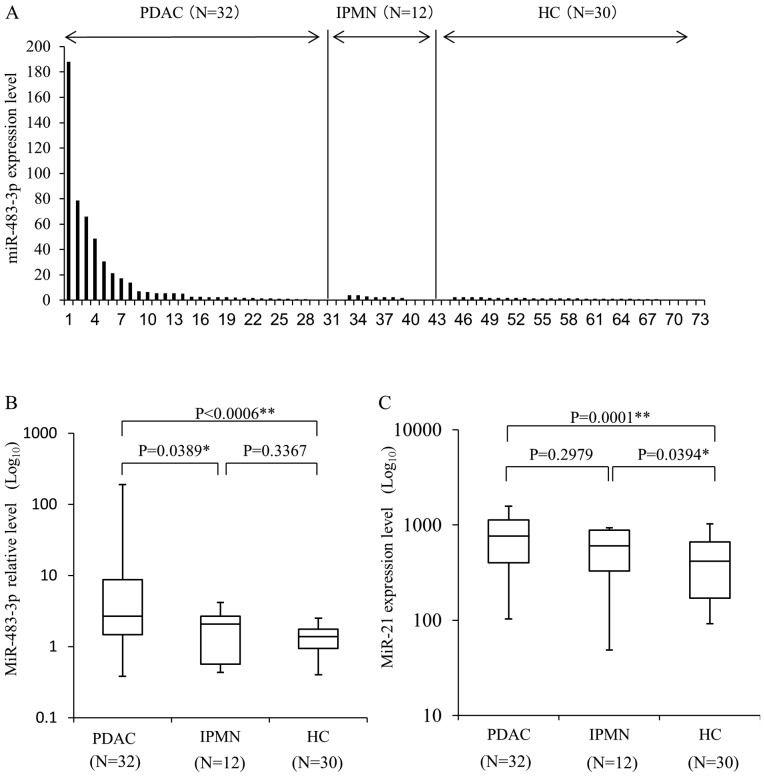
The expression of miR-483-3p and miR-21 in plasma samples. (A) The distribution of the plasma miR-483-3p expression level in all samples. (B) The miR-483-3p expression level in PDAC, IPMN and normal plasma samples. Boxes represent the interquartile range and the line indicates the median value. Whiskers indicate maximum and minimum values. miR-16 was used for normalization. The miR-483-3p expression level in plasma is significantly higher in PDAC than HC and IPMN group, while no significant difference is found between IPMN and the HC group. Mann-Whitney U test was used to evaluate statistical significance. (C) The plasma miR-21 expression level in PDAC, IPMN and HC. The plasma miR-21 level is significantly higher in PDAC than HC group, whereas no significant difference was observed between PDAC and IPMN. ^*^P<0.05 and ^**^P<0.01.

Next, we hypothesized that higher miR-483-3p expression in primary carcinoma cells would influence the plasma levels of miR-483-3p in PDAC patients. To determine the quantities of plasma miRNAs, we used cell-miR-39 as internal control miRNAs, and miR-16 was used for the normalization. Plasma miR-483-3p expression was calculated from the comparative Ct method by quantitative RT-PCR. Using this assay, circulating miR-483-3p was detectable in all plasma samples from 32 PDAC patients, 30 HC and 12 IPMN patients (Fig. 2A). The mean level of plasma miR-483-3p expression in the PDAC patients [16.50±6.49 (miR-483-3p/miR-16, mean ± SE)] was significantly higher than in HC (1.37±0.11, P=0.0006) and IPMN patients (1.90±0.39, P=0.0389) (Fig. 2B). We also evaluated the expression level of plasma miR-21, which was previously shown to be overexpressed in the plasma and tissue of PDAC (26,36–43). The mean expression level of plasma miR-21 (Fig. 2C) was significantly higher in the PDAC group [765.25±64.6 (miR-21/miR-16 ± SE)] than HC (414.54±44.7, P=0.0001). The plasma expression level of miR-21 in IPMN patients was also higher than in HC (P=0.0394), and no differences were seen between PDAC and IPMN (P=0.2979). While no difference was observed in the plasma miR-21 expression level between PDAC and IPMN patients, miR-483-3p expression in the plasma of PDAC patients was significantly higher than in that of IPMN patients. This result indicates that plasma miR-483-3p measurement has the potential to distinguish PDAC not only from healthy individuals, but also from the patient of IPMN.

### The utility of measuring miR-483-3p and -21 in plasma for discriminating PDAC from healthy controls and/or IPMN patients

**Figure 3:**
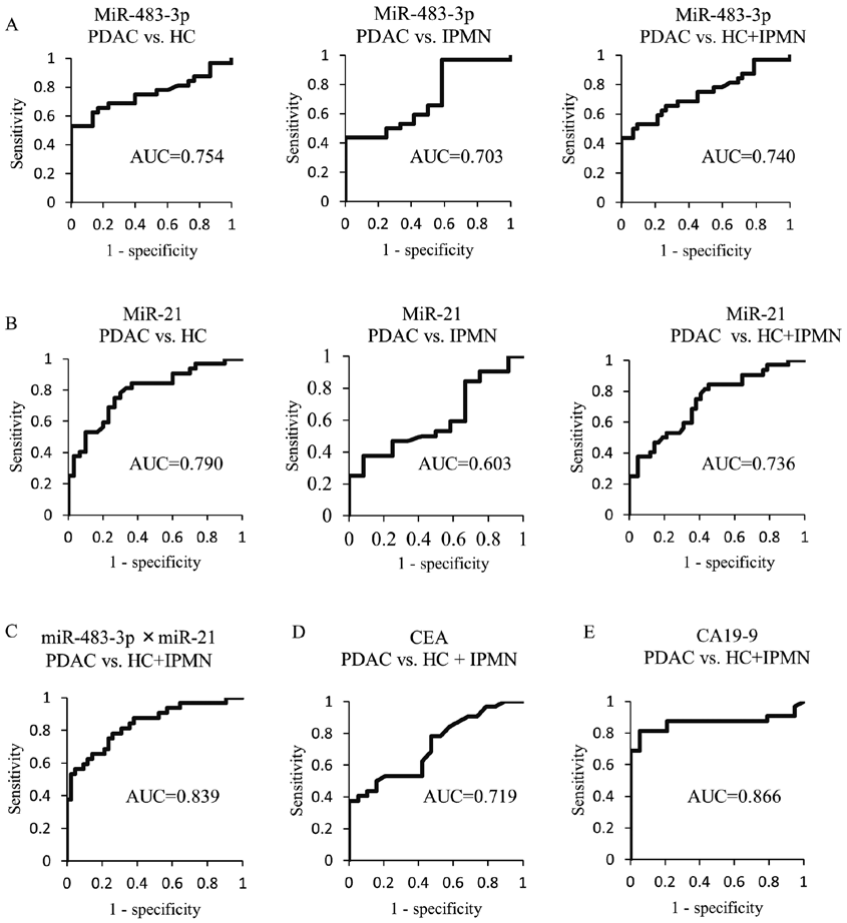
The ROC curve analysis of plasma miRNA and serum tumor marker levels for PDAC diagnosis. (A) The ROC curves for differentiating between PDAC and HC (left), PDAC and IPMN (center), PDAC, and HC and IPMN (right) based on the miR-483-3p plasma level. (B) The ROC curves for differentiating between PDAC and HC (left), PDAC and IPMN (center), PDAC, and HC and IPMN (right) based on the plasma miR-21 level. The ROC curves for differentiating PDAC from IPMN and HC based on the combination of the miR-483-3p and miR-21 plasma levels (C), serum CEA (D) and serum CA19-9 level (E).

To apply the expression value of plasma miRNAs for the diagnosis of PDAC, we then analyzed the ROC curves. The ROC curves for differentiating between PDAC and HC, and/or IPMN based on the expression level of miRNAs (miR-483-3p or miR-21) are shown in Fig. 3. The AUC of miR-483-3p (0.754) was slightly lower than that of miR-21 (0.790) for differentiating PDAC from HC. On the other hand, for discriminating PDAC from IPMN patients, the AUC of miR-483-3p (0.703) was higher than that of miR-21 (0.603), In addition, the AUC value based on the expression level of plasma miR-483-3p (0.740) and miR-21 (0.736) to distinguish PDAC patients from non-cancer individuals (HC and IPMN) was similar. Consequently, we compared the ability of measuring the plasma miR-483-3p and miR-21 to predict PDAC with that of the conventional tumor markers CEA and CA19-9. The respective AUC values of plasma miR-483-3p and miR-21 were higher than that of CEA (0.719), but lower than that of CA19-9 (0.866). Interestingly, the AUC (0.839) from the ROC curves for the combination of miR-483-3p and miR-21 was similar to that of CA19-9 for discriminating patients with PDAC from non-cancer individuals.

### The correlation between plasma miRNAs and clinicopathological factors

**Figure 4:**
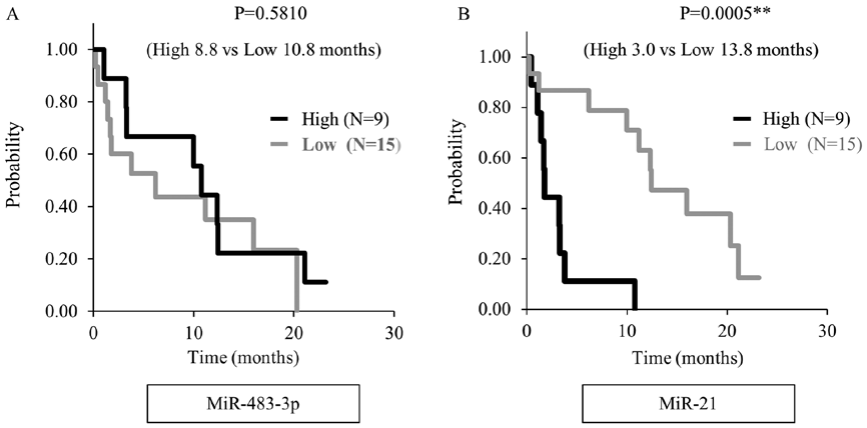
The correlation between plasma miRNA expression and overall survival (OS) of PDAC patients. Kaplan-Meier overall survival curve in 24 PDAC patients who were excluded based on their plasma miRNA expression. (A) There was no significant relationship between the plasma miR-483-3p level and overall survival. (B) The prognosis for PDAC patients was worse in those with high expression of plasma miR-21. ^**^P<0.01.

Finally, we evaluated the correlation between the plasma level of miRNAs and clinicopathological factors in 32 PDAC patients (Table III). We judged the miR-483-3p level as ‘high expression’ when it was higher than a cut-off value of 5. There was no significant relationship between the plasma miR-483-3p level and clinical factors with overall survival. The plasma miR-21 expression level was associated with advanced stage (P=0.023), metastasis to lymph node (P=0.007) and liver (P<0.001) when the cut-off value was defined as 850, as shown in Table IV. Moreover, overall survival was significant shorter in the high miR-21 expression group than in the low expression group when we analyzed by the Kaplan-Meier method in 24 unresectable PDAC patients, (3.0 months vs 13.8 months, P<0.001) (Fig. 4).

### Plasma miR-483-3p expression level is decreased after resection of PDAC

**Figure 5:**
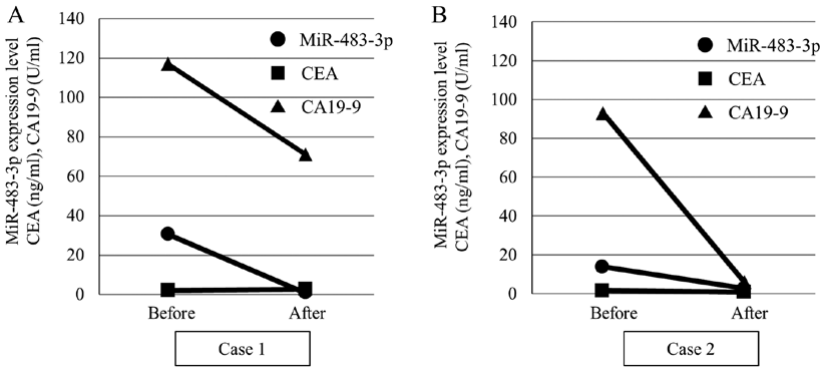
The plasma expression level of miR-483-3p and tumor markers before and after surgery. The plasma miR-483-3p level was drastically decreased after surgery in two PDAC patients: A, case 1 and B, case 2. Both cases were diagnosed as pancreatic adenocarcinoma and were classified as stage IIA (T3N0M0) using the UICC classification.

Since the plasma miR-21 level was not increased in operable patients (Table IV), we evaluated alterations in the plasma miR-483-3p level alone after resection of the tumor tissues. Fig. 5 shows representative cases of the plasma expression level of miR-483-3p and tumor markers before and after surgery. Case 1 was a 70-year-old female and case 2 was a 62-year-old female. In both cases, pancreaticoduodenectomy was performed for the diagnosis of pancreatic head cancer. By histological examination, the diagnosis was pancreatic adenocarcinoma and the classification stage IIA (T3N0M0) using UICC classification. As expected, a marked reduction of the plasma miR-483-3p expression as well as the CA19-9 value was observed after surgery, indicating that the expression of miR-483-3p was strongly associated with the presence of PDAC.

## Discussion

MiRNAs have been shown to play important roles in the tumorigenesis and/or development of various cancers. Since it exists stably in the plasma/serum, miRNA appears promising as a new biomarker for the diagnosis and treatment of cancer. In PDAC patients, numerous miRNAs including miR-21, miR-155, miR-196a and miR-210 have been detected in the plasma/serum, and have been suggested to be useful biomarkers for the diagnosis (6,7,9,26). In the present study, we examined the expression of miR-483-3p as well as miR-21 in the plasma of PDAC patient to assess whether these miRNAs would be useful markers for the detection of PDAC and for predicting the clinical status of this tumor. We clearly demonstrated for the first time that the plasma miR-483-3p level was significantly higher in PDAC patients compared to those of HC and IPMN patients and that the AUC value of miR-483-3p for discriminating PDAC from IPMN was higher than that of miR-21. miR-483-3p has been shown to promote cell proliferation through downregulation of its target gene, Smad4 in PDAC cells (42). Reportedly, the loss of Smad4 expression is frequently observed from the late stage of carcinogenesis of PDAC, but infrequently in IPMN (46,47). Taken together with our finding that miR-483-3p is expressed specifically in PDAC patients without showing a relationship to the clinical features, this miRNA may play a pivotal role in the carcinogenesis rather than the development of PDAC through reducing Smad4 expression.

Recent studies revealed that BD-IPMN is a risk factor for PDAC since the occurrence of PDAC during the follow-up period for BD-IPMN patients was not infrequent (48–50). To date, there are no highly sensitive methods to predict the occurrence of PDAC in IPMN patients. However, worsening diabetes mellitus and abnormal serum CA19-9 levels have been demonstrated to be useful to predict the development of PDAC in BD-IPMN patients (51,52), and the sensitivity of CA19-9 to predict concomitant PDAC was shown to be 45% (51). In the present study, the sensitivity of the plasma miR-483-3p level to differentiate PDAC from IPMN was 43.8%, similar to that of CA19-9, indicating that the evaluation of this miRNA in plasma could also be a useful tool for detecting the occurrence of PDAC in IPMN patients. The reduction of the plasma miR-483-3p level by surgical resection of PDAC may partially support this idea.

Overexpression of plasma miR-21 was demonstrated in PDAC and was correlated with a poor prognosis in this tumor (26,35–37). As had been shown in previous studies, we also demonstrated that the plasma miR-21 level was increased in PDAC patients compared to HC and associated with poor prognosis in unresectable PDAC patients. In addition, we revealed the relationship between the plasma miR-21 level and liver metastasis in PDAC patients. The involvement of MiR-21 in the metastasis of PDAC cells was also clarified *in vitro*. Moriyama *et al* showed that miR-21 precursor transfection promoted proliferation, invasion and gemcitabine resistance in PDAC cells *in vitro* (30). Moreover, Kadera *et al* reported that expression of miR-21 in tumor-associated fibroblasts as well as PDAC cells enhanced the metastatic potential (33). These findings suggest that stromal cells as well as metastatic PDAC cells are likely to be the origin of plasma miR-21, and thus high plasma levels of this miRNA are detected in patients with advanced stage PDAC.

Intriguingly, the AUC value for the diagnosis of PDAC was increased to a level similar to that of CA19-9 when the miR-483-3p and miR-21 plasma levels were combined. This is due to mutually independent mechanisms that enhance the expression of the respective miRNAs. As discussed above, miR-483-3p and miR-21 are upregulated in the process of carcinogenesis and development, respectively. Thus, the evaluation of miR-483-3p together with miR-21 would forecast the status in patients with PDAC.

## Figures and Tables

**Table I:** Experimentally validated circulating miRNA in PDAC.

miRNAs	Authors/(Ref.)	Expression in PDAC
miR-21	Wang, *et al* (26), Liu, *et al* (34), Ali, *et al* (35), Liu, *et al* (36), Kong, *et al* (37)	Upregulation
miR-210	Wang, *et al* (26), Ho, *et al* (28), Liu, *et al* (36)	Upregulation
miR-155	Wang, *et al* (26), Kon, *et al* (37)	Upregulation
miR-196a	Wang, *et al* (26), Liu, *et al* (36), Kong, *et al* (37)	Upregulation
miR-221	Kawaguchi, *et al* (29), Ali, *et al* (35)	Upregulation
miR-200a, 200b	Li, *et al* (14)	Upregulation
miR-18a	Morimura, *et al* (27)	Upregulation
miR-181a,181b	Liu, *et al* (36)	Upregulation
miR-20a, 24, 25, 99a, 185, 191	Liu, *et al* (34)	Upregulation
miR-1290, 24, 134, 378, 146a, 484, -628-3p, 1825	Li, *et al* (53)	Upregulation
let-7d, miR-146a	Ali, *et al* (35)	Downregulation

**Table II:** The characteristics of PDAC patients, IPMN patients and healthy controls (HC).

	PDAC (N=32)	IPMN (N=12)	HC (N=30)
Age			
Mean ± SD (range)	70.6±8.7 (48–89)	74.6±8.8 (61–89)	44.5±19.1 (21–85)
Gender			
Male	22	6	11
Female	10	6	19
Stage^a^	I/II/III/IV	1/8/10/13	

a UICC classification.

**Table III:** The association of miR-483-3p expression level with clinicopathological factors in PDAC.

	Low expression (N=18)	High expression (N=14)	P-value
Age			
<70	8	8	0.476
≥70	10	6	
Gender			
Male	12	10	0.773
Female	6	4	
Diabetes mellitus			
Yes	9	8	0.688
No	9	6	
Location			
Head	7	7	0.530
Body-tail	11	7	
Tumor size (mm)			
Mean ± SD	45.6±22.0	38.3±12.6	0.424
T classification^a^			
T1–T2	7	8	0.305
T3–T4	11	6	
N classification^a^			
Yes	5	4	0.960
No	13	10	
Liver metastasis			
Yes	5	4	0.960
No	13	10	
Ascites			
Yes	2	2	0.788
No	16	12	
Stage^a^			
I–III	10	9	0.618
IV	8	5	
Operation			
Yes	3	5	0.217
No	15	9	

a UICC classification.

**Table IV:** The association of miR-21 expression level with clinicopathological factors in PDAC.

	Low expression (N=22)	High expression (N=10)	P-value
Age			
<70	13	3	0.127
≥70	9	7	
Gender			
Male	14	8	0.355
Female	8	2	
Diabetes mellitus			
Yes	11	6	0.599
No	11	4	
Location			
Head	10	4	0.773
Body-tail	12	6	
Tumor size (mm)			
Mean ± SD	38.8±15.8	50.3±22.5	0.137
T classification^a^			
T1–T2	12	3	0.197
T3–T4	10	7	
N classification^a^			
Yes	3	6	0.007^c^
No	19	4	
Liver metastasis			
Yes	2	7	<0.001^c^
No	20	3	
Ascites			
Yes	2	2	0.387
No	20	8	
Stage^a^			
I–III	16	3	0.023^b^
IV	6	7	
Operation			
Yes	7	9	0.186
No	15	1	

a UICC classification.

b P<0.05;

c P<0.01.
